# The Nursing Supplement

**Published:** 1890-01-18

**Authors:** 


Being the Extra Nursing Supplement of "The Hospital" Newspaper.

All Contributions for this Supplement should be addressed to the Editor, The Hospital, 139-140, Salisbury Court, Fleet Street, London, E.C., and should have the word "Nursing" plainly written in left-hand top corner of the envelope.

### En Passant.

WIDELY READ.—A matron writes: "I enclose a short account of our Christmas tree. I do not know whether you care to insert it, but here it is. After the notice of my appointment to this hospital had appeared in your columns, I had congratulatory letters from many old friends in the nursing profession from all parts of the world, which proved to me how widely The Hospital is circulated, and how it may form a connecting link between those who have worked together for a time, but whose busy lives prevent much correspondence."

NURSING AT EDINBURGH.—The annual report of the Edinburgh Royal Infirmary makes special reference to the nursing department. It says: "Miss Fergusson continued to superintend all departments of the hospital, visitors and patients alike testifying to the efficient way in which everything was managed. The managers again expressed their great satisfaction at the able and efficient manner in which Miss Spencer, zealously aided by her assistant superintendents, had discharged the very important duties of the nursing department. The training school attached to it continued to gain in popularity and usefulness, as evidenced by the fact that of the sixty-eight trained nurses on the staff on October 1, 1888, twenty-two had left within the year. The vacancies thus caused had been satisfactorily filled. During the past year the number of applicants desirous of joining the training school exceeded that of any previous year since its establishment, while the vacancies to be filled had been somewhat fewer than usual." The necessity for a nurses' home is also brought forward, and is likely to receive attention this year.

SOUTH HANTS INFIRMARY. — A new nursing scheme is before the committee of the Royal South Hants Infirmary, which we hope will be carried through. A report has been prepared by a sub-committee, in accordance with the directions of the Committee of Management, in which a series of suggestions are set forth based on the adoption of a system of nursing by probationers. The future nursing staff is recommended to be composed in this way: Six head day nurses at £30 per annum, to be raised at the end of two years' service to £35—any further increase to be at the discretion of the Committee of Management; one night superintendent at £35; one out-patient nurse at £25, not to exceed £30; eight one-year probationers paying a fee of thirty guineas by half-yearly payments in advance, and an entrance fee of one guinea; six two-year probationers paying an entrance fee only of one guinea. The distribution of these fourteen probationers would be ultimately as follows: Six on day duty, six on night duty, two supernumerary for extra or special work; the night duty to be taken in turn by all. With the introduction of probationers an addition of five extra servants would be necessary—viz., kitchen-maid, sewing-maid, and three housemaids. There are two points which will require consideration in this scheme—first, the appointment of a trained lady superintendent and the arrangement of lectures for the nurses; and secondly, whether it would not be better to have fewer one-year probationers. We are sorry to say some of the committee oppose the scheme, though the present style of nursing is at least twenty years behind the times, and has given rise to numerous complaints.

SHORT ITEMS.—The nurses of the Evelina had a pleasant little dance last week.—Lady Verney has given a bust of her sister, Florence Nightingale, to the Aylesbury Infirmary.—A correspondent from Montreal who is now advertising for nurses in our pages, says there is a great lack of trained nurses in Canada.—A writer to the *Tablet* points out that change of religion is not a sufficient ground for the termination of a contract with a nurse, and that damages can be obtained for wrongful dismissal under such circumstances.

THE NURSE'S-CASE BOOK.—This useful little book can now be obtained by sending a postal order for one shilling and a penny stamp to the publisher of The Hospital. The Case-Book contains twenty-two pages, and each page admits of a fortnight's record of a patient's condition, including temperature, pulse, etc., and notes on the treatment, history, and ultimate result of the case. The book is so small it can easily be carried in the apron-pocket, and ought to prove specially useful to probationers who desire to keep a private record of their cases for their own instruction.

GRUMBLERS.—We publish a letter this week from two Peterborough nurses who do not seem to appreciate the difference between a confirmed grumbler at everything all round, and the nurse who comes forward with a specific complaint. Our strictures of the 4th inst. were called forth by two letters which we did not print, but from which we quoted enough, we thought, to display in what an uncharitable spirit they were written. We protest against this spirit amongst a few in defence of the whole body of nurses, who, we are sure, do not echo such unkind and uncalled-for remarks.

NURSING THE LEPERS.—Referring to the recent announcement that a lady was about to start from London as nurse for the leper colony at Molokai, the London correspondent of a Sheffield paper says: "Being desirous of ascertaining the name of the heroine who had thus elected to consign herself to a life-martyrdom and probably to a dreadful death, I communicated with the vicar of the metropolitan parish in connection with whose local mission the lady is going out, for further information. The reply was that 'the nurse who is going out to the lepers in Molokai wishes to be unknown.'"

THEIR LITTLE BEST.—It is amusing to find out that in one little corner one little person is still going on shrieking forth at the Pension Fund. All the hysterical spite of this one little person cannot hurt, or even be heard, by the Pension Fund authorities, still we wish to "nail to the counter" one statement which affects this paper. We have *not* omitted to publish the report of the Pension Fund, the Chairman's summary of which will be found in our issue of October 19 last. As for other statements concerning the finances of the fund which are being screamed in that same little corner, they are too absurd to be worth contradicting. The accounts of the fund are audited, and our great City financiers who are on the committee are surely better judges of how the accounts are audited than this little nameless person, who is apparently working for a fit of apoplexy. Oh, for "one small touch of charity" to try and raise those who

Strain to make an inch of room
For their sweet selves, and cannot hear
The sullen Lethe rolling doom
On them and theirs and all things here.

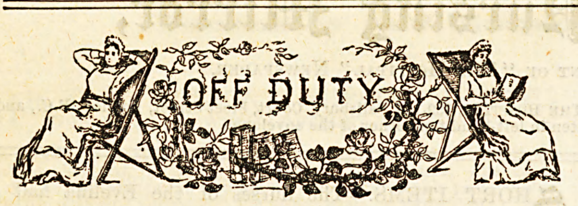

### Mr. Trantum's Hobby.

HE was retired from the Navy—H.M. Navy. Anyone could find that out in less than ten minutes' talk with Mr. Trantum. The enthusiastic, almost passionate, love for his profession; the gentle scorn for all "tubs" that ride the ocean outside the pale of the service; the courtly, knightly devotion to women and little children, are all hall-marks not to be mistaken. But it was many a day since Mr. Trantum trod the decks of a man-of-war. Things were slightly different then to what they are now, and the old sailor looked out from his nook, christened The Bunk, with screwed-up, suspicious eyes upon the deadly improvements of to-day. Not that he was outwardly captious. He did not assure his hearers half-a-dozen times in an hour that the service was "going to the devil, sir," like old Major Jones-Smythe, at the other end of the little seaside village, incessantly did. He was too loyal to the old profession for that; the discipline ingrained in his nature kept him from carping, albeit he was oftimes a little bewildered by the strides of science.

Mr. Trantum was a bachelor—a peaceful state for which he thanked the Lord daily. Specially was he grateful for that beatific condition when the fact that he was an uncle forced itself upon his consideration, and he had to ponder over the troublous life of Jack, his brother in the artillery, who had a wild struggle to get along, partly because of impecuniosity, partly because of a trial in the person of Mrs. Jack. Mr. Trantum adored ladies as a body—individually, they were inscrutable compositions to the simple-hearted sailor, and if he took frivolous, flighty Mrs. Jack, of the artillery, as a criterion, small wonder that womankind was beyond his ken. But he understood children—there was no mistaking that fact; and the little folk knew he did. There's no need to point out to either children or dogs their friends.

"Send me down little Jack," wrote he, at once, when he heard of the tiny lad's wearisome illness, "send him here; the smell of the sea will build him up, if anything can."

The boy was the weakly one of an uproariously strong family; he was always puling and fretting; always the one, above all others, to fall downstairs and out of windows; always the first to catch whatever disease was flying about.

"He's that contrairy he does it a-purpose," declared Betts, the good woman who filled Mrs. Jack's place to her children while the still pretty mother careered through all the Woolwich gaieties; "he's just a plague, Johnny, he is; there!"

So, when the boy showed symptoms of a graver sort than sprained ankles and twisted shoulders, nobody was at all surprised, and the Jack Trantums were only too thankful to send him off to his uncle, thus shifting the responsibility from their own heedless shoulders.

"God bless my soul!" Mr. Trantum stood gazing down at a white-faced, human morsel, whose travelling wraps had been removed.

"He do look a poor thing, sir," spoke up Betts. "He's bin and done it this time, he 'as. He's run through most every complaint, and now nothin' would suit him, but he's catched a decline, and——"

"There, there!" Mr. Trantum gently waved his hand to stem the torrent. "My good woman, hush—h!" and Betts was as speedily as possible hustled back to her other charges at Woolwich. Then Mr. Trantum set to work.

"I must build the little chap up bit by bit. Please God I'll succeed."

The Bunk was a queer, old-fashioned, overgrown cottage, half way up the cliff, for, of course, Mr. Trantum lived, and meant to die, with his face to the blue sea, as a true sailor should.

"Now, little man, we must have your days spent down on the beach, and Ben shall carry you up home."

"Who's Ben?" asked the small new-comer.

"My man-of-all-work, to be sure. Never heard of Ben Bromich, hey? He and his daughter Jane are the establishment; she cooks, he waits. Hi! Ben! Come here and pay your respects to the Woolwich Infant!"

"Sarvint, young sir!" was the hale old retainer's welcome.

"What d'ye think of him, Ben?"

"They don't seem to make 'em so big as they did in my time, sir," answered Ben after a pause, and a puzzled look of compassion crept over his brown, wrinkled face as he regarded the atom, all eyes and skin, perched upon a chair. "Do'ee ail, little master?" he gently enquired.

"Oh, no," was the shrill reply; "I'm all right. I've only got a cold, so I've come down here to live with my uncle, and pick up a bit." A violent fit of coughing ended the breathlessly-spoken speech, causing the two old men to look anxiously at each other.

"Darter Jane," said Ben Bromwich, "'tain't likely that little shaver will weather the storm; he's doomed."

"Afeard he be, feyther," said middle-aged Jane, as she cut her father's bread and butter. "Master had best send him back. He doesn't know what a 'sponsible thing a child is, specially a child as anybody can see is took for death."

But the master thought otherwise. Had little Jack been as other children the old gentleman might have wearied of him in time, but he was not. Quaint and old-fashioned, his comical, incessant, pattering chatter was irresistibly fascinating to his uncle. And the pluck of the morsel was a special charm. There was nothing Jack did not want to attempt, panting and fighting for breath meanwhile.

"If so be, sir, as you'd shut him up in bed," said Darter Jane, "I'd keep linseed on him night and day. It'd be a sight more to the purpose than hevin' you and feyther a-carryin' of the blessed child down and a-heavin' of him up that there cliff day after day."

"Don't be sich a fool, Darter Jane," put in her father, politely; "leave young sir to me and the master. We're buildin' of him up, that's what we're a-doin', but 'tain't no use explainin' things to wimmen-folk," he added resignedly.

*(To be continued.)*

### The Nurses' Bookshelf.

### A MANUAL OF NURSING, MEDICAL AND SURGICAL.*

THE question is often asked whether a male or female teacher is best adapted for instructing probationer nurses. No doubt both have special merits of their own, and in small institutions, where a matron or superintendent of nurses is thoroughly alive to her work, and has a special capacity for conveying her knowledge and experience to others, it would be folly to replace her by a member of the sterner sex, who in all probability knows little or nothing of the domestic duties of nurses. On the other hand, the requirements of the training schools, apart from mere technical knowledge, render it obligatory that the nurse should possess a quasi-scientific interest in her art, which is best acquired from an official medical authority. To meet this want, it has been more or less the practice in most training schools, the custom originating with the Nightingale Fund Council at St. Thomas's Hospital, for the resident medical officer or some junior member of the staff to deliver a course of lectures to nurses on medical and surgical subjects, elementary, of course, but bearing on the scientific aspect of the diseases which they are likely to meet with. The outcome of these arrangements has been a series of

* By Laurence Humphry, M.A., M.B., etc. London: Charles Griffin and Co., Exeter-street, Strand.

text-books or manuals of varying merit from the two classes of instructors, and there can be no question that nurses should avail themselves of the best from the two sources. Mr. Humphry's book is the latest addition to the scientific series, and we can confidently recommend it to nurses as an excellent guide and companion. The manuals written by Dr. Wallace Anderson, of Glasgow, and by Mr. Joseph Bell, of Edinburgh, on medical and surgical nursing respectively, are excellent in their way, but Mr. Humphry deals with both subjects in smaller compass, besides being up to date, and including monthly nursing, massage, and other specialities. Preparatory to a brief description of the more important diseases, which are divided into sections, or, say, stems, each being presumably the subject of a separate lecture, there are explanatory notes of the anatomy and physiology of the various organs involved, which are also represented by a series of wood engravings. The illustrations are very numerous and cannot fail to enhance the value and popularity of the manual. Beside the above they comprise temperature charts of the more common febrile disorders and a variety of sick-room appliances, methods of bandaging different parts of the body, and the book finishes with an appendix of receipts on invalid cookery, which every nurse ought to know something about. We would advise all nurses to possess a copy of the work.

### Keeping Christmas.

At **Nottingham General Hospital** there was the usual Christmas tree, from which presents were distributed to 150 patients. The members of the medical and nursing staffs assisted in the distribution, which was carried out under the direction of Miss Rimington, the matron. The wards were prettily decorated, and in the evening a concert was given, Nurse Wood being amongst the performers.

At **Belvedere Hospital, Glasgow**, plenty of toys were given to the children, and the nurses had a most enjoyable concert and dance in the evening. Dr. James W. Allen gave an address, in which he described the improvement in the hospital since Mrs. Sinclair was appointed matron. Formerly there were only two nurses to each ward. They alternately took night and day duty, and were on an equal footing. There was no period of probation—an applicant was engaged and put on full duty at once. Her merits and defects were discovered sooner or later. There was no special training, no instruction. The nurses cooked and ate their meals in the fever wards. The nurses are now systematically taught in the wards and in the lecture-room, and, after examination, are granted a certificate of proficiency. There are three nurses in each ward—a head nurse, day nurse, and night nurse. The probationer signs for two years on being engaged, and advances in the service as she is found worthy of confidence. The nurses now take their food in a cheerful dining-hall, away from the atmosphere of the fever ward.

At the **Fleming Memorial Hospital** there was a splendid tree. The generous founder of the hospital—Mr. John Fleming—after a short address to the little patients, distributed the prizes, and the pleased expression of the children as they received their presents with exclamations of delight formed a sight no less gratifying to those adults present. The visitors were provided with tea, and a musical entertainment, at which the inmates sang, was given. The lady superintendent and her nurses had put up some very pretty decorations.

At **Bradford Infirmary** a special dinner was served on New Year's Day, and on the Thursday the children had two Christmas trees. The presentation of the toys was preceded by a few remarks from the Mayor, who took the opportunity of heartily thanking all those ladies and gentlemen who had made Christmas presents to the infirmary, and of also thanking the nurses for the efforts they had made in decorating and giving a bright and cheerful appearance to the wards. Later in the evening a very successful concert was given in the Rand Ward by the choir of St. Paul's Church, Shipley.

The annual treat to the patients of the **East London Hospital for Children, Shadwell**, has been given, and besides the present inmates about 100 old patients were invited. Before leaving the hospital each child was presented with some warm clothing and a New Year's present. The Rev. E. Bray, chaplain to the hospital; Rev. Dr. Carter, Rev. T. F. German, Dr. Eustace Smith, and others attended.

At **Bedford General Infirmary** the patients held Christmas one night, the servants the next, and the nurses the third night. It was amusing to see Dr. Shelding, Miss Newberry, and the nurses waiting on the servants on the second night; but truly it can be said that at Bedford everyone had their share of the fun. Mr. Tyrer presented the infirmary with a splendid musical box.

The Christmas and New Year's arrangements at **Horton Infirmary, Banbury**, this year have been of rather a more lively description than hitherto. The usual Christmas dinner of roast beef and plum pudding, etc., was given to those patients who were allowed to take the old English fare, and this was supplemented by Mr. C. H. David's annual present of dessert to each patient. After dinner each patient received a Christmas letter, kindly given by Mrs. Richard Hadland, Great Bourton; also one from Miss Norris, Shanklin; and, in addition to this, the same lady sent a useful present to everyone living in the hospital. In the afternoon the Rev. C. E. Graham-Jones, with his choir boys, enlivened the wards with the singing of Christmas carols. On New Year's Eve a musical entertainment was given under the direction of the house surgeon, and a Christmas tree was tastefully decorated and illuminated under the supervision of the matron and her staff. Each patient and resident in the hospital received a useful present from it. The donations for distribution among the patients consisted of clothing of every description, useful articles for men and women, and toys, etc., for the children, which were contributed by the ladies of the neighbourhood.

The annual address was given to the nurses of **Glasgow Royal Infirmary** on New Year's Day. In the course of his speech Mr. Hugh Brown referred to the fact that Miss Ross had been appointed to an important position at the Victoria Infirmary, and he was glad to say that the vacancy thus caused had been filled from the nurses in the institution. Miss Thomas had been promoted to fill Miss Ross's place, then Miss Bowman was promoted to fill that of Miss Thomas, and Nurse Alexander had been promoted to the post formerly filled by Miss Bowman. It was a great satisfaction to the directors that they were able to fill the vacancy from among their own nurses, and it was, besides, an encouragement to the other nurses. The directors were always anxious to give promotion where it was deserved, and it had always been to him a matter of regret that when vacancies occurred in other places their best nurses were taken from them. He had also to notice the removal of Miss Thorn to Ayr, and he was glad to learn that she was giving the utmost satisfaction.

The theatricals at **St. Bartholomew's** were excellently arranged and excellently acted, the costumes being specially good in "The Critic." A cantata, "The Wreck of the Hesperus," was sung, and loudly applauded by a large audience. The red plush drop-curtain perhaps excited as much interest and admiration as any other feature of the entertainment. The usual apologies had to be made for those of the performers who were suffering from influenza.

At **Ancoats Hospital, Manchester**, the matron, Miss Chambers, was able to give useful gifts to every patient, nurse, and servant. On January 7, the annual entertainment took place in the large ward of the Albert Victor Wing; there was music and recitations, but the chief event was the waxworks, in which the doctors, secretary, and nurses took part. Refreshments were served by Miss Chambers to nearly 200 visitors.

The annual Christmas treat was given to the patients at the **Central London Throat and Ear Hospital** on Friday last, when a large number who had been inmates during the past year assembled with those who were resident in the hospital at the present time. Over a hundred people, including many children, sat down to tea at five o'clock. After the tables had been cleared amusements of many kinds were heartily entered into by all present. A huge Christmas tree was perfectly laden with toys of every description, thanks to the liberality of Messrs. Lazarus and Rosenfield and to the editor of *Truth*, as well as to the matron of the hospital, who had provided many useful articles for the older people. After the presents had been given away, Mr. Charles Bertram, the wellknown legerdemain, gave his inimitable entertainment of conjuring, etc., to the immense delight of everyone. The Royal Punch and Judy Show also gave much enjoyment. Several ladies and gentlemen kindly rendered assistance in the musical portion of the entertainment. Miss Edith Lumley and Miss Rigge presided at the piano. The wards were all very tastefully decorated and illuminated, and the greatest credit is due to Miss Danter, the deservedly esteemed matron, for the success of the entertainment.

### Everybody's Opinion.

[*Correspondence on all subjects is invited, but we cannot in any way be responsible for the opinions expressed by our correspondents. No communications can be entertained if the name and address of the correspondent is not given, or unless one side of the paper only be written on.*]

### OVERWORKED NURSES.

Peace with Honour writes: In your issue of 28th ult. (December) you make reference to Leith Hospital, and I enclose you several cuttings from the Edinburgh *Evening News*, showing the Leith Hospital nurses have all struck against such oppression. You will also notice a most disgraceful remark, which has been made public in the letter by "Justice," *i.e.*, the praising of the matron of this hospital at the expense of the characters of the doctors and the poor nurses. As the nurses have done nothing wrong, the directors ought immediately to find out who "Justice" is, and have a suitable punishment put upon such a cowardly individual.

Two Peterborough Nurses write: Surely it is a breach of chivalry, not to say a breach of faith, after inviting nurses to state their grievances, straightway to turn round upon them, and call them grumblers and (practically) liars. To the initiated, the expression of those grievances, as set forth in your pages, has been singularly moderate, carrying probability on the face of it. But if a nurse's word be unreliable, it follows as a logical deduction that a matron's is untrustworthy, every trained matron having been a nurse originally. A statement of existent facts, in answer to a direct query, for instance, "off-duty" on alternate days, is not precisely a complaint. Many of us believe the fact, not the statement of it, to be an economic error, an injury alike to employer and employed. Will you not consider that the old adage, "Like master, like man," holds good of a matron and her nurses. We, at any rate, have neither been dismissed for insubordination, nor charged with being grumblers and untruthful. Miss Heanley's nurses have no grounds for duplicity or dissatisfaction. We can confidently refer you to her judgment, as we can also furnish satisfactory testimonials from our present hospital authorities. Hoping you will see good cause to modify your somewhat sweeping condemnation, true, doubtless, in the sense of all men being liars, we should be glad could some reason be brought forward for exacting a perfection incompatible with human infirmity, and unlooked for in any other calling from the nursing sisterhood.

### Presentation.

On Saturday last the nurses of the Great Yarmouth Hospital presented their late house surgeon (E. Caudwell, M.R.C.S., L.R.C.P.) with a handsomely bound album, bearing his initials and a suitable inscription, as a token of their esteem and regret at his departure.

### Appointments.

[It is requested that successful candidates will send a copy of their applications and testimonials, with date of election, to The Editor, The Lodge, Porchester-square, W.]

Kent County Asylum.—It is with great pleasure we record the appointment of Miss Marion King as assistant matron of this asylum at Barming Heath. Miss King trained at King's, and then went to Chester for fever nursing; was sent from there to cases at Paris and Brussels, and for the last year has acted as night superintendent at Great Ormond-street. We have always maintained that a trained nurse should have charge of asylum attendants, so as to instruct them in the simplest rules of nursing, and we are delighted that anyone with Miss King's great knowledge should take that knowledge where it is so much needed.

### Notices.

In view of certain changes which are imminent in consequence of the large and ever-increasing circulation of The Hospital, the Editor requests every matron, sister, or nurse who takes the paper regularly to send him notice of her name and address, to The Lodge, Porchester-square, London, W. Postcards may be used.

The Exhibition of Nursing Uniforms has been invited to Cardiff, Swindon, Shrewsbury, Sheffield, and other places, but we are unable to enter into any further engagements for our dolls till we see how they bear their journey to Liverpool and Glasgow.

### Vacancies.

**Matrons.**

Dartmouth Cottage Hospital, £35.
South-Eastern Fever Hospital, £100.

**Sister.**

Bolton Infirmary.

**Nurses.**

Bath Royal Hospital Home, £25.
Birkenhead Nurses' Institute.
Bournemouth Sanatorium, £25.
Brompton Cancer Hospital, £25.
Cambridge Home for Nurses.
Canterbury Infirmary, £25.
Cardiff Infirmary.
Central London Throat Hospital, £20.
Chelsea Infirmary, £18.
Cheltenham Institution.
Croydon Hospital (two).
Devonport Albert Hospital, £25.
Dorset County Hospital.
Fitzroy House Home Hospital.
General Nursing Institute.
Grimsby Nursing Home.
Hampstead Home Hospital, £20.
Hitchin Nursing Institution.
Hollond Institution, Nice.
H. M. Hospital, Stepney, £25.
Kilmarnock Infirmary.
Levick Institution, Paris.
Liverpool Bluecoat Hospital, £25.
Montreal, Canada, £37.
Newark Hospital, £25.
Newcastle-on-Tyne Home.
Norfolk and Norwich Hospital.
Norwich Nurses' Home.
Nottingham Nursing Association, £25.
Nursing Institution, 96, 97, 98, Wimpole-street, London, W., Mr. Wilson has several vacancies for thoroughly-trained nurses.
Pendlebury Children's Hospital, £20
Ryde Nurses' Home.
Salford Infirmary, £27 10s.
Southport Infirmary (two).
South London Institute.
St. Leonards-on-Sea.
St. Olave's Union (three), £20.
Stroud Hospital, £23.
Swansea Institution, £25.
Ventnor Consumption Hospital, £20.
Victoria Park Hospital, E., £20.
Weston-super-Mare Hospital, £25.
Windsor Infirmary, £20.

**District Nurses.**

Edinburgh Jubilee Institute.
Great Bedwyn, Wilts., £25.
Pontarfran, Brecon.
Winchcombe Rural Nursing Association.

**Probationers.**

Birmingham Children's Hospital.
Boston Hospital.
Bristol Hospital for Children and Women.
Durham County Hospital.
Guildford Hospital.
Hull Royal Infirmary.
Kent Nursing Institution.
Newark Hospital.
Newport Infirmary.
Nottingham Nursing Association.
Workhouse Infirmary Association.

### Amusements and Relaxation.

**N.B. FOURTH QUARTERLY WORD COMPETITION**
**Commenced Jan. 4th, 1890, ends March 29th, 1890.**

**Three Prizes of 15s., 10s., 5s., will be given for the largest number of words derived from the words set for dissection.**

**Proper names, abbreviations, foreign words, words of less than four letters, and repetitions are barred; plurals, and past and present participles of verbs, are allowed. Nuttall's *Standard* dictionary only to be used.**

**N.B.—Word dissections must be sent in WEEKLY, arranged alphabetically, with correct total affixed.**

**The word for dissection for this, the THIRD week of the quarter, being "INFLUENZA."**

**Results of First Week.**

| Names. | Jan. 9th. | Totals. | Names. | Jan. 9th. | Totals. |
|---|---|---|---|---|---|
| Holland | 30 | 30 | Qu'appelle | 41 | 41 |
| Red Cape | 35 | 35 | Whitehoe | 30 | 30 |
| Nurse Marie | 27 | 27 | Nurse Clague | 40 | 40 |
| Garfield | 39 | 39 | Lauriston | 48 | 48 |
| Midget | 40 | 40 | B. E. T. | 16 | 16 |
| E. M. S. | 40 | 40 | Jacko | 33 | 33 |
| Sister | 41 | 41 | Lady Clara | 39 | 39 |
| Duchess | 39 | 39 | Patience | 43 | 43 |
| Ellice | 46 | 46 | Ecila | 41 | 41 |
| Reldas | 41 | 41 | Maude | 40 | 40 |
| Jenny Wren | 38 | 38 | Esperance | 41 | 41 |
| Mopus | 37 | 37 | Ruby | 41 | 41 |
| Neptune | 40 | 40 | M. W. | 40 | 40 |

**Notice to Correspondents.**

**N.B.—All letters referring to this page which do not arrive at 139 and 140, Salisbury-court, London, E.C., by the *first post on Thursdays*, and are not addressed PRIZE EDITOR, will in future be disqualified and disregarded.**

